# Factors associated with older adults' perception of health risks of hot and cold weather event exposure: A scoping review

**DOI:** 10.3389/fpubh.2022.939859

**Published:** 2022-11-10

**Authors:** Priyanjali Ratwatte, Helena Wehling, Sari Kovats, Owen Landeg, Dale Weston

**Affiliations:** ^1^Behavioural Science and Insights Unit (BSIU), UK Health Security Agency (UKHSA), Porton Down, United Kingdom; ^2^Climate Change and Health Unit, UK Health Security Agency (UKHSA), Porton Down, United Kingdom; ^3^Behavioural Science and Evaluation, Health Protection Research Unit (BSE HPRU), London, United Kingdom; ^4^Environmental Change and Health, Health Protection Research Unit (ECH HPRU), Chilton, United Kingdom; ^5^Department of Public Health, Environments and Society, London School of Hygiene and Tropical Medicine (LSHTM), London, United Kingdom; ^6^Extreme Events and Health Protection, UK Health Security Agency (UKHSA), Porton Down, United Kingdom

**Keywords:** risk perception, health protection, heatwaves, cold snaps, public health, older adults

## Abstract

**Introduction:**

Hot and cold weather events are increasingly becoming a global burden resulting in premature and preventable morbidity and mortality, particularly in vulnerable groups such as older people and people with chronic health conditions. However, risk perception regarding weather is generally poor among vulnerable groups which often acts as a barrier to the uptake of critical health-protective behaviours. A more cohesive understanding of determinants of risk perception is needed to inform public health risk communication and behaviour change interventions that promote protective health behaviours. This scoping literature review aimed to understand factors influencing perception of personal health risks in vulnerable groups as a result of exposure to hot and cold weather events.

**Methods:**

A five-stage scoping review framework was followed. Searches were run across Medline, PsychInfo, Web of Science and EMBASE. Papers were included if they provided rationale for risk perceptions in vulnerable groups in indoor/domestic environments and focussed on samples from OECD countries.

**Results:**

In total, 13 out of 15,554 papers met the full inclusion criteria. The majority of papers focused on hot weather events: one study exclusively examined cold weather events and one study addressed both cold and hot weather events. Included papers focused on older adults aged 65+ years. The papers identified eight factors that were associated with older adults' personal health risk perception of hot and cold weather events: (1) Knowledge of the relationship between hot/cold weather and health risks, (2) presence of comorbidities, (3) age and self-identity, (4) perceived weather severity, (5) Beliefs associated with regional climate, (6) past experience with weather, (7) misconceptions of effectiveness of protective behaviours, and (8) external locus of control.

**Conclusions:**

Future research should explore risk communication methods by implementing the identified risk perception determinants from this review into health protection interventions targeting older adults. Further understanding is needed regarding risk perceptions in non-elderly vulnerable groups, for examples individuals with chronic diseases or disabilities.

## Introduction

Hot and cold weather events, including heatwaves and cold spells, can pose significant risks to health (1–4). The frequency and intensity of heatwaves is increasing globally, due to anthropogenic climate change (5) and heat-related risks in particular were identified as key risks to health and wellbeing in the 3rd UK Climate Change Risk Assessment (6), ultimately changing exposure and models of care (e.g., care at home and digital health) (7).

While it is difficult to identify individuals at a heightened risk of death from heat, several groups have been determined to be more vulnerable to heat-related mortality. These include older adults, above 65 years of age, due to problems with thermoregulation as a result of ageing (8), people with pre-existing illnesses such as cardiovascular and respiratory diseases, diabetes and Parkinson's disease (8), and those whose ability to perform self-protective behaviours is impaired (e.g., dementia, mental illness) (9). In summer 2020, three heatwaves in England caused 2,556 deaths (excluding COVID-19 deaths), the highest heat-related mortality since the introduction of the Heatwave Plan for England in 2004, and the majority of reported deaths (*n* = 2,244) occurred in adults 65+ years (10). Furthermore, cold-related mortality is a significant problem for the UK population, particularly for older adults and people with chronic illnesses, and has been linked to an increased risk of stroke, heart attack and injury (11–15). Overall, the majority of cold- and heat-related illnesses and deaths are considered premature and preventable (15–18).

Despite the established risk of hot and cold weather, research suggests that at-risk populations do not view themselves as such. For example, one study indicated that over half of older adults aged 75+ years do not view themselves as vulnerable to hot and cold weather risks (19). Similarly, another study found that 31% of individuals with a heart condition and 28% with a lung condition did not perceive themselves as at risk from heatwaves (20). The well-established relationship between risk perception and behaviour demonstrates the importance of ensuring that vulnerable groups appropriately recognise the risk to themselves and understand the appropriate mitigating factors (21). Indeed, alongside under-estimating their risk, individuals aged 65+ years have demonstrated poor adoption of recommended protection behaviours (such as opening windows, drinking fluids, avoiding physical activity as hottest part of the day, and dressing warmly, keeping windows closed, and maintaining boilers during periods of cold weather (22). Similarly, it is known that individuals with chronic long-standing illnesses often do not consider hot weather as a risk to their health and subsequently they are unlikely to adapt their behaviours in response to heat (23). Furthermore, many UK residents, including individuals from vulnerable groups, hold positive attitudes towards hot weather and therefore, may be less likely to perceive themselves as at risk and to perform protective behaviours (23, 24).

Considered together, the evidence suggests that it is important to increase the uptake of recommended behaviours to mitigate the negative health impacts of hot and cold weather events on vulnerable groups in the UK. Some research has identified that some vulnerable groups, such as older adults, do not associate themselves as vulnerable and therefore, do not view themselves as intended recipients of hot-weather risk-communication interventions (25). As a first step, it is important to further explore why vulnerable individuals tend to not perceive themselves as being at risk, and to identify methods of increasing risk perception within these groups. This scoping literature review aimed to assess and synthesise the evidence on factors that are associated with perception of personal health risks from hot and cold weather events which to our knowledge has not been undertaken in a comprehensive manner to date. The findings of this review could be pertinent for future public health messaging, by providing recommendations for ensuring a more appropriate perception of risk within vulnerable groups in the UK by addressing relevant underlying barriers in this population.

## Methods

A preliminary scope of the literature identified that the existing evidence base on investigating perception of health risks of hot and cold weather in vulnerable populations varies in terms of data analysis methods and result reporting. Additionally, the fact that most papers utilised qualitative-design methodologies makes comparability and synthesis of findings challenging. For this reason, this study utilised a five stage scoping review framework which allows the comprehensive examination of an emerging research domain (26) and is appropriate for addressing research areas that lacks consistency in quality of evidence and methods. While systematic reviews tend to focus on the randomised control trial as the gold standard of research design, this framework provides techniques suitable for synthesising qualitative data.

### Stage 1: Identifying the research questions

The review aimed to answer the following questions:

1. How do vulnerable populations perceive personal health risks to hot and cold weather?
2. Which factors specifically affect/influence or are associated with risk perception to the negative health impacts of hot and cold weather event exposure?

### Stage 2: Identifying relevant papers

This review originally utilised a two-step search strategy. To survey the existing literature, a preliminary search was conducted on Medline and PsycInfo to identify papers examining how older adults and individuals with chronic illnesses or disabilities perceive personal health risks to hot and cold weather events, examining titles, abstracts, subject terms and keywords of relevant papers. This was conducted to familiarise the literature informally and identify appropriate terminology to utilise in the second more extensive search. A second literature search was conducted on 1st February 2021 across four databases: Medline, Embase, PsyInfo and Web of Science using terms related to ‘risk perception” and “risk awareness” (contextual search terms) and; hot and cold weather (threat/risk search terms) (see Supplementary material for full list of search terms) that were formulated into Boolean search algorithms (see Supplementary material for search database search strategies). The search was limited to papers that were published in English and included research examining the risks of indoor or domestic exposure. Vulnerable populations have a tendency to spend the majority of their time indoors, compared to the general population (27, 28) and therefore, it is important to consider the role of indoor domestic environments in prevention measures in relation to extreme weather and changing models of care will mean increased care provided in the home setting. Furthermore, the review focused on OECD country participant samples due to assumed similarities of population characteristics and response systems that would be comparable to the target populations in the UK and response systems. This aims to identify and understand potentially similar factors that may undermine public health interventions, taking into account the particular needs of target vulnerable groups.

As the initial search was conducted in February 2021, a follow-up search was conducted on 12th September 2022 to identify relevant literature published since. Due to time constraints, the research team conducted a condensed search on Google Scholar of the first 20 pages of the results, using the following search terms:

“risk perception” OR “threat perception” AND heat OR “hot weather” OR heatwave OR “Heat wave” OR cold OR “cold weather” OR “cold spell”.

### Stage 3: Study selection

Papers were first title and abstract screened by a single researcher. Papers were excluded for only measuring risk perception and not exploring rational of risk perception, not providing focused observations on risk perception for vulnerable groups but instead on a broader participant sample in terms of age and vulnerability characteristics, focusing on health protection behaviours and not risk perception and only investigating knowledge of health risks vs. personal perception of risks (see Table 1 for full list of inclusion and exclusion criteria). Full-text versions of the papers were subsequently reviewed by two researchers to judge inclusion/exclusion. Papers during the title and abstract screening stages were not reviewed by a second researcher due to time limitations. For the updated Google Scholar search, two members of the research team title, abstract and full-text screened papers.

**Table 1:** Full list of inclusion and exclusion criteria.

**Inclusion**	**Exclusion**
Participant sample includes persons 65+ years of age or persons with long-term health conditions or disabilities.	Participant sample does not include persons 65+ years of age or persons with long-term health conditions or disabilities.
Examines/discusses perception of personal health risks of exposure to extreme hot or cold weather hazards in household/domestic environments.	Participant sample from clinical and care home settings.
Examines/discusses relationship between perception of risk to extreme hot and cold weather hazards and resultant health behaviours.	Discusses risk communication methods or mortality risk of extreme hot and cold weather hazards with no exploration of risk perception.
Participant sample from OECD countries (*list of OECD countries*).	Discusses health risk perception of extreme weather or climate change broadly with no exploration of health risk perception of extreme hot and cold weather.
Primary data.	Conference proceedings/abstracts/short reports/reviews/commentaries.
	Participant sample from non-OECD countries.
	Articles not in English.
	Articles not related to “human”

### Stages 4 and 5: Charting the data and collating, summarising and reporting the results

Papers that met our criteria were analysed using content analysis (29) in order to identify relevant insights in relation to factors that are associated with how older adults perceive personal health risks to hot and cold weather. Firstly, data were extracted by reading through individual findings, identifying the presence of related words and concepts which were used to generate themes and to code responses into a descriptive numeric summary. Extracted data relating to study characteristics were then tabulated according to author(s), year of publication, country of publication, study aims, type of weather event, study design/methods and participant characteristics.

Data extracted from papers included from the updated Google Scholar search were treated the same as the original database search and were analysed to identify whether they fit into existing themes or if new themes were present.

## Results

**Figure 1:**
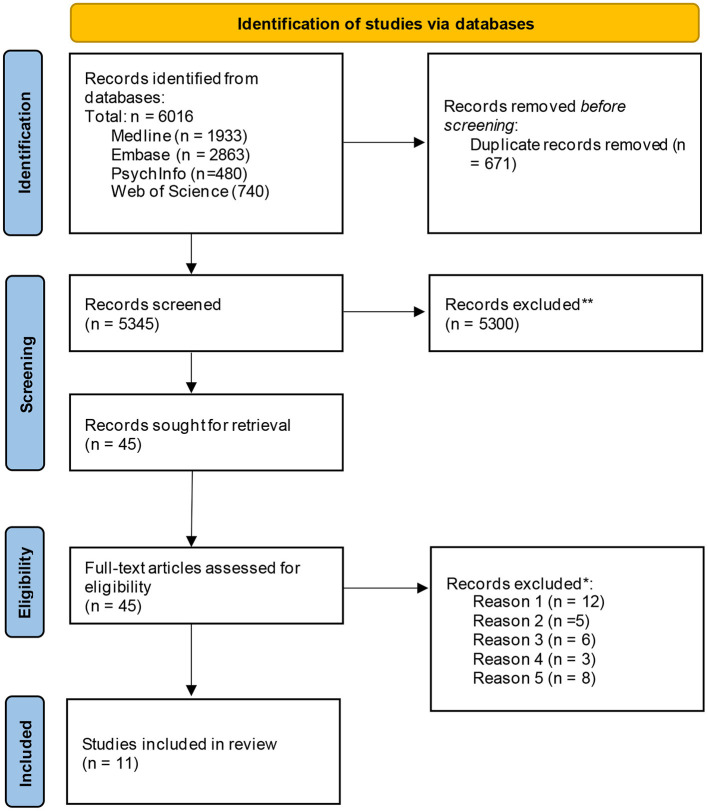
PRISMA flow diagram of paper selection process.

Overall, 6,016 papers were identified through the original database search. After the removal of 671 duplicates, 5,345 papers were title and abstract screened and 5,300 articles were excluded during this phase. A total of 45 papers were progressed for full-text screening and were reviewed by a single reviewer, and 12 of the papers were reviewed further by a second reviewer to assess fit (see Table 1 for full list of inclusion and exclusion criteria). At this stage, papers were primarily excluded because they only measured risk perception but did not explore what influences it; focused on vulnerable groups that were not older adults or individuals with chronic illnesses or disabilities (e.g., geographical location and vulnerability, low socioeconomic status); examined a broad population cross-section and did not provide specific insight into target vulnerable groups; only focused on intention to perform behaviours vs. understanding perception of risk and; only investigated knowledge/awareness of health risks and did not explore risk perception. A total of 11 papers met the full inclusion criteria and these papers were progressed for data charting and analysis (see Figure 1).

**Figure 2:**
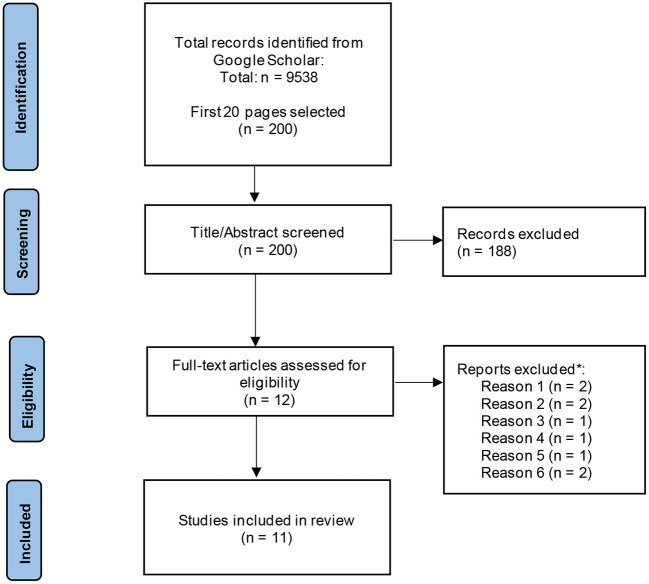
PRISMA flow diagram of paper selection process from follow-up search on Google Scholar.

Considering the inclusion and exclusion criteria used for the original search, 9,538 papers were identified during the updated Google Scholar search. Of these 9,538, the first 20 pages (200 hits) of study titles were screened, resulting in the exclusion of 9,516 papers. Abstract screening produced 12 papers which were progressed to full-text screening, which resulted in a total of 2 papers that met the full inclusion criteria (see Figure 2).

### Study characteristics

In relation to the types of vulnerable groups represented in the included papers, all papers sampled only older adults. Only two studies investigated individuals with chronic illnesses or disabilities within their participant sample (23, 30); however, the results did not describe risk perception specific to this population.

Of the 13 included papers, three included participant samples with a broad age range including older adults (55–94 years) (23, 25, 31–38) while the majority focused exclusively on older adult populations (*n* = 8) (30, 39, 40). Older adult participants across nine of the included papers lived alone (i.e., non-institutionalised), while the remaining two papers included participants who stayed in retirement or nursing home dwellings (32, 36). A total of five studies utilised qualitative interviews to examine risk perception and six papers used a mixed method design; a combination of qualitative interviews and focus groups and questionnaires.

In terms of weather types examined in the papers, the majority focused exclusively on hot weather (heatwaves, *n* = 9, 81%) (25, 30–32, 34–37, 39, 40). Two papers focused on cold weather: one on cold snaps (33) and one investigated both hot and cold temperatures in summer and winter periods (37).

Table 2 provides a summary of the study characteristics, including publication year, country of publication, study aims, weather event, design/methodology and participant sample of the included papers.

**Table 2:** Summary of the characteristics of the included papers including publication year, country of publication, study aims, weather hazard, study design/methods and participant sample characteristics.

**Publications**	**Year**	**Location/Country**	**Aims**	**Weather event**	**Design/Method**	**Participant Characteristics (No. of participants, age range in years)**
Abrahamson and Wolf (25)	2009	Norwich, UK	Investigate knowledge of health risks; Investigate perception of health risk; Identify health protection/adaptive behaviours	Heatwave	Qualitative semi-structured interviews	*n* = 73, 72–94
Banwell et al. (31)	2012	Sydney, Australia	Identify health protection/adaptive behaviours	Heatwave	Qualitative: pamphlet field trial; Focus groups and; interviews.	*n* = 20, 65+
Beckmann and Hiete (30)	2020	Augsburg, Germany	Identify predictors of heat health risk and willingness to adapt behaviour	Heatwave	Mixed methods measuring: health risk perception, locus of control and heat risk knowledge.	^*^n = 72, 65–74
Bittner and Stosel (32)	2012		Investigate perception of health risk; Identify health protection/adaptive behaviours	Heatwave	Qualitative semi-structured interviews (43 items)	*n* = 20, 64–94
Erens et al. (23)	2021	England, UK	Investigate perception of health risk; Identify health protection/adaptive behaviours	Heatwave	Mixed methods: survey and focus group	*n* = 1,872, 18–74
Gascoigne et al. (33)	2010	Loughborough, UK	Intervention development	Cold snap	Qualitative: advice booklet field trial; semi-structured interviews.	*n* = 22, 65–87
Kemen et al. (38)	2021	Cologne, Germany	Investigate perception of health risk; Identify health protection/adaptive behaviours	Hot weather	Qualitative- interviews/questionnaires	*n* = 258, 65–93
Lane et al. (39)	2014	New York, USA	Investigate impact of knowledge of health risks on health protection behaviours	Heatwave	Mixed methods: survey examining AC access, heat-illness prevention behaviours and awareness of heat-health warnings; focus groups on heat-health knowledge and behaviours in older adults and caregivers.	*n* = 38, 65+
Loughnan et al. (34)	2014		Investigate knowledge of health risks; Identify health protection/adaptive behaviours	Heatwave	Mixed methods: Semi-structured interviews; health behaviour diary; 7-point Thermal Comfort Scale ASHRAE.	*n* = 40, 55+
Mattern et al. (35)	2000	Philadelphia, USA	Investigate perception of health risk	Heatwave	Mixed methods (6-month community-based study): education sessions, provision of marked thermometers, and provided heat-related manual. 24-iten questionnaire measuring perceptions of barriers to heat related morbidity; administered preintervention and 8 weeks post intervention.	*n* = 34, 65–85+
Richard et al. (40)	2011		Investigate impact of risk perception and health beliefs on protective behaviours	Heatwave	Mixed methods: Cross-sectional interview study, 169 close-ended item questionnaire measuring Health Belief Model constructs; self-report measure of air conditioner use; 36 physical and mental health subscales.	^*^n = 185, 60–80+
Valois et al. (36)	2020	Quebec, Canada	Investigate impact of health beliefs on protective behaviours	Extreme heat	Mixed methods: Qualitative interviews and 71 closed-ended questionnaire.	*n* = 1002, 64–85+
Wolf et al. (37)	2010	Norwich, UK	Investigate perception of health risks	Heatwave & cold spells	Qualitative interviews.	*n* = 15, 75–83

* Reports data specific to older adults within a larger sample with a broader age range.

### Perception of risk

Risk perception amongst the papers was understood as the degree to which an individual believed they were personally susceptible or vulnerable to the health-related risks of hot/cold weather. Older adults either did not perceive themselves as being at risk of experiencing illness as a result of hot or cold weather exposure or, they believed that personal risks to health of exposure was low. Abrahamson and Wolf (25) reported that only a small proportion of older adults in their study viewed hot weather as “challenging”, despite participants being classified as objectively vulnerable. One study that involved a health promotion intervention (35) that was designed to improve heat risk awareness in older adults reported that the proportion of participants that were “unconcerned about heat-related illness” increased from 62% (pre-intervention) to 76% (post-intervention). Interestingly, Kemen et al. (38) identified that the a large proportion of their sample of older adults viewed themselves as moderately at risk of hot weather exposure (42.6%) and one third (31.6%) identified as “clearly” at risk/extremely challenging (high risk), whereas a minority of the sample viewed themselves as being at little to no risk (25.2%).

#### Factors associated with risk perception

Table 3 illustrates the incidence of factors associated with risk perception that were identified and categorised *via* content analysis across the reviewed papers. The factors will be described in more detail in the following sections.

**Table 3:** Presence of identified factors influencing risk perception in older adults across included papers.

**References**	**Knowledge of the relationship between hot/cold weather and health risks (*n* = 5)**	**Presence of Comorbidities (*n* = 6)**	**Age and self-identity (*n* = 6)**	**Perceived weather severity (*n* = 4)**	**Beliefs associated with regional climate (*n* = 6)**	**Past experience with weather (*n* = 6)**	**Misconceptions of effectiveness of protective behaviours (*n* = 10)**	**External locus of control (*n* = 5)**
Abrahamson and Wolf (25)	X	X	X		X	**X**	**X**	
Banwell et al. (31)				X	X	X	X	
Beckmann and Hiete (30)	X	X						X
Bittner and Stosel (32)		X		X			X	
Erens and Bob (23)				X	X		X	
Gascoigne et al. (33)	X		X		X	X	X	
Kemen et al. (38)		X	X				X	
Lane et al. (39)	X	X	X		X	X		
Loughnan et al. (34)				X			X	X
Mattern et al. (35)						X	X	X
Richard et al. (40)	X		X					
Valois et al. (36)						X	X	X
Wolf et al. (37)		X	X		X		X	X

##### Knowledge of the relationship between hot/cold weather and health risks

Possessing knowledge of the health-related risks of hot and cold weather amongst older adults often did not explicitly increase risk perception (25, 30, 33, 39, 40). Two papers suggested that this low perceived risk equally applies to older adults who possess poor knowledge about weather event exposure (33, 40).

##### Presence of comorbidities

The presence of existing comorbidities such as respiratory illness and cardiovascular disease was associated with an increased perception of personal health risk in relation to exposure to hot and cold weather (25, 30, 32, 37–39). However, it is important to note that some papers suggest that not all older adults with comorbidities consider or recognised that they are at a higher risk of experiencing health-related symptoms as a result of hot and cold weather events (25, 30). For example, the participants in a qualitative methods interview study (37) believed that the heightened risk was only associated with the presence of comorbidities and not advanced age. Furthermore, two other studies found a lack of knowledge amongst some participants of how specific medication treatments for comorbidities could further exacerbate health risks of hot weather exposure for example as a result of not being unaware of the influence of thermoregulation from administering specific medications (25, 39).

##### Age and self-identity

There was a prominent belief amongst older adults that increasing age is not associated with increasing physiological vulnerability (25, 33, 37–40). Four out of five papers which identified this factor reported that older adults who were aware of the associated health risks as a result of hot and cold weather event exposure were able to identify other older people as vulnerable (e.g., people living alone with “less support”, housebound people, “unwell” or “frail” people that are over the age of 70 years). However, these study participants did not self-identify as being vulnerable/at increased risk despite being within the same age bracket (25, 33, 37, 39). Wolf et al. (37) found that participants described other people from the same age range, 75–83 years of age, as being more “old” or “frail” than themselves (33, 37).

##### Perceived weather severity

Individuals' personal perception of the severity of hot and cold weather events was a key factor associated with personal risk in a number of papers (31, 32, 34). More specifically, participants in a qualitative interview study (32) observed that participants had a greater trust in ‘one's own senses' when judging the severity of temperature vs. utilising objective assessment tools, such as thermometers to determine risk. The theme of trusting personal feelings/senses over advice/information was also observed in a qualitative focus group study which observed that participants were aware of the importance of hydration to combat hot weather severity but were unconcerned about not meeting recommended water requirements because they did not feel thirsty often (23). Similarly, according to a mixed methods study involving semi-structured interviews, the use of diaries and questionnaires (34), participants preferred to guide their beliefs and behavioural responses according to their own experiences vs. scientific advice on weather and climate change. Indeed, it was further suggested that the older adults in this study tended to be sceptical of climate change which might additionally explain their lowered perception of heat severity. This observation was also identified in a qualitative study involving interviews and focus groups where participants rejected beliefs about global warming and climate change because their personal experiences of heat contradict scientific evidence relating to increasing temperatures being caused by climate change (31).

##### Beliefs associated with regional climate

Several papers highlight the influence of the local climate in specific regions and countries and resulting beliefs on older adults' personal risk appraisal of hot and cold weather (23, 31, 33, 37, 39). Two qualitative studies from Australia observed a normalisation of heat resulting in lowered perceived heat risk: One study found that older adults believed they had acclimatised to heat, attributing their self-claimed acclimatisation to having lived with heat all their lives. Bearing hot weather had therefore, become a part of their identities with some participants even suggesting genetic adaptation due to prolonged exposure to heat from previous generations (31). The second study identified that older adults disregarded the health risks of heat by rationalising that heat was a normal and mostly enjoyable experience of summer in Australia (34). Several studies conducted in England found that participants viewed themselves as at low risk of heat because they enjoyed hot weather (25, 38). According to several other studies, older adults did not see themselves at risk because they expected mild summer and winter periods, (23, 33, 37, 39). More specifically, participants in one study viewed hot weather in the UK as a rare occurrence and this resulted in a separation between risk and self, due to the underlying belief that hot weather is not a concern in the UK (37). A different sample of older adults from the USA assessed the severity of the hot weather by comparing local summers to tropical regions. Participants stated that ‘nothing happens', implying that they do not feel hot and only a few days during the summer period were viewed subjectively as significantly hot (39). Preparation for mild or average cold weather was also observed in the included literature, with participants viewing winter periods from their past as being more severe (33).

##### Past experience with weather

Relatedly to the previous factors on individual weather perceptions and cultural appraisal of weather, the findings from four papers suggest that a lack of presence of illness or strong discomfort (e.g., dizziness, faintness, sunburn) during hot and cold weather periods, either in the past few years prior to the study or throughout the lifespan, lowered older adults' perceived risk during current weather events (25, 31, 33, 39). This was rationalised by participants' belief that weather conditions were more severe in the past, either because hot or cold weather felt subjectively worse or that fewer coping resources were available in the past (e.g., lack of centralised heating to address cold weather). As a result, participants felt they had acclimatised and were more resilient to hot and cold weather events which prevented them from becoming ill despite their advanced age (31, 33). Furthermore, older adults are more likely to perceive risk to hot and cold weather events as less severe if they do not know any family or friends who had experienced illness as a result of these types of weather events (35, 36).

##### Misconceptions of effectiveness of protective behaviours

Although the majority of older adult participant samples across the included papers did not perceive themselves as being at risk to hot and cold weather events, specific health-protective behaviours were performed with the intention to alleviate discomfort in response to hot weather exposure (23, 25, 31–37). Commonly performed behaviours in response to hot weather included: minimising time spent outdoors, drinking more fluids, consuming light meals and cold foods and drinks, using fans, blocking direct light by closing doors, blinds or curtains, wearing light clothing, cooling the body with water and minimising physical activity. Commonly reported behaviours in response to cold weather were: dressing warmly when going outdoors and when indoors, using hot water bottle, turning on radiator and sealing sources of draught. The prevalent belief was that older adults would adapt daily behaviours to “accommodate” or “adapt” to heat, that these behaviours were intuitive or “common sense” behaviours, and that it was not necessary to change behaviour beyond this to address potential health risks (23, 25, 31–33, 35–37). Two mixed method study (35) observed that older adult participants believed that the behaviours adopted to accommodate or adapt to the discomfort associated with heat could completely diminish potential health risks when objectively they are not adequate; performing these behaviours fostered a false sense of security for example, the use of air conditioning, eliminated the need to resort to any alternative behavioural strategies to cope with heat (34). The older adult participants in Erens et al. (23) believed specifically that only adopting one single behaviour, in this case staying indoors, was sufficient in order to eliminate the risks of a heatwave. Furthermore, participants in this study were more concerned about protecting themselves against the risks of sun damage to the skin, believing that ageing has ‘thinned' the skin, rather than the impact of heat itself. Contrastingly, a different paper observed that their sample of older adults on average adopted 8–10 coping behaviours for hot weather, and that a higher number of adopted coping behaviours employed was associated with a higher perceived risk of hot weather (38).

##### External locus of control

Some of the literature illustrates that older adults attribute the occurrence of hot and cold weather events to external factors as they are viewed as ‘naturally occurring' events (30, 34–37). The response to the perceived uncontrollable nature of these types of weather events was stoic (35, 37): adopting beliefs such as having to ‘put up with' hot and cold weather and choosing to not be concerned about occurrences which they lack control over. The results of another mixed methods study (36) demonstrated that promoting behavioural responses to hot weather with focus on an internal locus of control resulted in increased perception of health risks and increased intentions to adapt to heatwaves in older adults. In contrast to these findings, an ANOVA and regression model in one study found an association between an external locus of control and risk perceptions, suggesting that the more a person is convinced that the incidents happening in their life are based on fate or accident, the more they perceive heat as being a risk (30).

## Discussion

This scoping literature review aimed to identify factors associated with vulnerable populations' perception of personal health risks as a result of being exposed to hot and cold weather events. The identified literature focuses primarily on older adults (65+ years of age) due to a lack of insight into specific findings in regard to other vulnerable groups. Overall, the evidence suggests that older adults do not perceive themselves as being at increased risk of experiencing health risks as a result of hot or cold weather event exposure, which is consistent with a recent evaluation of the England heatwave plan (19). The majority of findings from this review on risk perception are relevant to hot weather events as the majority of included papers focused on hot weather. The review identified a total of eight factors associated with personal health risk perception of hot and cold weather events in older adults: knowledge of the relationship between hot/cold weather and health risks, presence of comorbidities, age and self-identity, perceived weather severity, past experience with weather, misconceptions of effectiveness of protective behaviours, and external locus of control.

The studies generally highlight that many older adults do not believe that increasing age is associated with increasing physiological vulnerability to hot and cold weather because they may not sufficiently consider the relationship between advanced age and heightened risk, (33, 40). However, a lack of awareness or acceptance may not provide a comprehensive explanation for low perception of health risks in this population and possessing an understanding of health risks does not necessarily mean that individuals perceive themselves to be at risk (25, 30, 39). Moreover, reinforcing people's understanding of the relationship between advanced age and heightened health risks may not result in improved risk perception due to the negative connotations associated with being at risk as being “elderly” or “old”, held by some older adults. Indeed, participants viewed other people that they classified as “old” as being at risk but did not self-identify as “old” despite falling within the same age category, therefore describing groups who are at increased risk as “older people” does not work as this description does not match their self-perceptions (37). The dissonance of this statement may result from a rejection of associations of frailty with being elderly. Previous research has identified a tendency for older adults to see themselves as younger than their chronological age (41). Individuals who feel subjectively younger in comparison to an average person of the same age tend to have higher old age boundaries, meaning a shift towards not classifying themselves and others of this age group as old, and therefore at self-classifying as not being at an increased risk (42).

On the other hand, the presence of comorbidities that heighten health risks of exposure to hot and cold weather events was associated with an increased perception of risk (25, 30, 32, 37–39). However, the review identified a disparity between older adults whom acknowledged and understood how their comorbidities heightened their personal health risks (25, 30, 32, 37, 39), and others who lacked knowledge of this heightened risk, e.g., unaware of influence of thermoregulation from administering specific medications (25, 39). Additionally, it is not well understood if increased risk perception due to the presence of comorbidities translates to the performance of appropriate health protection behaviours.

The following findings focus on messaging in advertising but may be transferrable to a certain extent to a public health messaging context. Older adults' perceived age is considered an important consideration and should portray individuals in an active way that fits into their own perceptions about themselves (43). Particularly, emphasis is given to avoiding to portrayals as them as being “sick, poor and powerless” as findings shows they are more active and have less anxiety and concern about ageing. This finding is further supported by a recent study examining older adults' responses to the Covid-19 pandemic suggests that they have a desire to distance themselves cognitively from the heightened threat to older adults, by adopting a subjectively younger age (44). This understanding can help avoid evoking particular stereotypes in messaging which might be rejected by older adults, for example as these could offend cognitively younger-older adults (43). Similarly, researchers have concluded that self-perceived age may influence adoption of health protection behaviours, however this aspect has not been explored in the context of heat related behaviours and risks (45, 46).

The present review additionally identified that knowledge and beliefs about the health risks of exposure to hot and cold weather may be related to older adults' past experiences of these weather types (25, 31–36, 39). Some older adults have a tendency to rely on personal judgements on the severity of hot and cold weather in assessing health risk perception, and subsequently referred to their personal experiences of hot and cold weather from young-age (in which individuals likely had better health), transferring these past experiences to the present, without sufficiently considering the disparity of their current advanced age. Cultural norms that have been cultivated by past experiences of hot and cold weather, contributing to beliefs about acclimatisation, may also have a negative impact on perception of health risks (31, 33).

A culture of looking forward to hot weather due to perceived rarity of occurrence is prevalent in several countries which reportedly leads to lowered risk perception due to a desire to embrace hot weather (23, 31, 33, 37, 39). Furthermore, as people expect and subsequently prepare for mild hot and cold weather in summer and winter periods, they subsequently undermine risk and may be underprepared to respond to health risks. Such perception could be problematic as it contradicts to the actual weather data that suggests an increase of extreme hot weather periods particularly, especially in the recent years (47). Future research should continue to examine whether the recent increase in heatwave has had an impact on people's beliefs on the likelihood of experiencing extreme weather events. Furthermore, cultural norms which undermine risk awareness and protective behaviours need to be addressed in future interventions and public communication tools.

Despite older adults believing themselves to be at low risk, the intention to change behaviours was primarily motivated by a desire to alleviate discomfort from heat exposure or to minimise skin damage from UV exposure, rather than responding to health risks in the first place (23, 31, 32, 35, 36). The literature suggests that achieving comfort is a primary motivation for behaviour as opposed to risk alleviation. Furthermore, it has been evidenced that individuals are less likely to agree on the effectiveness of protective behaviours if they do not perceive themselves to be at risk (19). This could further explain the preference to perform low-impact behaviours that result in immediate positive consequences, e.g., physical relief, over more impactful behaviours, which focus on health risks. However, one study observed that older adults who performed several protective behaviours had a higher risk perception, implying that more behaviours are being performed to minimise risk (38). It is therefore important to further understand people's motivations for engaging in behaviours in response to extreme weather in order to re-frame their reasoning and decision-making regarding performance of more impactful risk reduction behaviours in addition to less impactful, comfort-oriented actions.

The present literature demonstrates mixed results as to whether risk perceptions are associated with an internal or external locus of control. The finding that some older adults view hot and cold weather events as exclusively externally controlled suggests a belief there is little opportunity for them to respond proactively by engaging in protective behaviours (30, 34–37). It is unclear to what extent these attitudes are stoic or apathetic. A stoic approach could be supported by the ideas expressed in the ‘past experiences' factor in that older adults view themselves as being able to endure these types of weather events due to accumulated experience. On the other hand, the presence of an apathetic orientation could be explained by the view of some health protection behaviours as “common sense” behaviours, possibly indicating a dismissal of the severity of the potential health risks (25, 31–33, 35–37). In contrast, one paper suggests that external, rather than internal locus, is associated with higher risk which contradicts the findings from the remaining five papers. However, it is important to note that these findings only demonstrated a weak correction, which may undermine this observation, particularly given that this provides a unique finding amongst all six papers in this specific area. Non-etheless, future research should aim to provide more detailed evidence to help clarify the precise relationship between locus of control and risk perceptions in older adults. However, according to one study (36) demonstrates the potential of reframing individuals' locus of control to improve risk perception by promoting an internal locus of control of behavioural responses to heatwaves, which could result in an increased perception of health risks and increased intentions to adapt behaviour.

Finally, rather than exclusively relying on the vulnerable individual in adopting of protective behaviours, adopting a whole systems approach that involves central and local governments, the healthcare sector and communities, could result in improved recognition of individuals who might be at increased risk of health impacts from extreme weather events and dissemination of appropriate interventions and/ or care plans. An example is the development and commissioning of clinical assessment tools, such as the frailty index in older adults, which can be used to inform recommendations targeting potentially modifiable factors, such as ensuring adequate home heating, therefore emphasising the need of housing interventions in this context (48).

## Limitations

Several limitations should be noted when interpreting the findings of this literature review. Firstly, this review did not include unpublished or grey literature. Due to the varied design methodologies featured across reviewed papers, it was not possible to assess for scientific quality however this is permissible within the scoping literature review methodology. The majority of included papers focused on hot weather events, with only a single paper exclusively examining risk perception of a cold weather event. Therefore, caution must be exercised when applying the identified factors to understand risk perception of cold weather events. While this review set out to examine how older adults and individuals with chronic illnesses and disabilities perceive personal health risks to hot and cold weather event exposure, the reviewed literature only contained specific insights focusing on older adult participant samples. Furthermore, it was not clear to what extent the included older adults were also recorded as people who have chronic illnesses and/or disabilities in the data. Finally, it was not possible to differentiate risk perception amongst different age categories of older adults as most of the included papers generalised observations across the entire age range of 65–95+ years. Therefore, future research may be needed to understand whether there are meaningful differences between those at lower and those at the higher end of the age spectrum that will need to be considered for risk communication strategies.

## Conclusions and future directions

This scoping literature review concluded that a large proportion of older adults do not perceive themselves as being at risk of experiencing negative health impacts as a result of hot and cold weather event exposure. Risk perception of hot and cold weather exposure is known to be associated with a range of factors which can undermine the success of risk communication strategies. The present findings suggest that future research should explore interventions and risk communication methods that aim: to address the misconceptions and biases in relation to health risks that older adults may possess, including the relevance of age identities and past experiences; to stress the importance of performing protective behaviours for older adults who have comorbidities that heighten their health risks; to acknowledge the positive appraisal of hot weather in intervention development; to investigate how risk perception of hot weather may change as the frequency, intensity and duration of heatwaves increase; to highlight additional benefits to risk reduction of the performance of specific behaviours such as alleviating discomfort and; to increase internal locus of control of the performance of health protection behaviours.

## Author contributions

PR developed the exclusion and inclusion criteria with input with DW, SK, and OL. PR and DW assessed the extracted abstracts and assessed all included papers. PR and HW wrote the paper. DW, SK, and OL commented on drafts of the paper. All authors read and approved the final manuscript.

## Funding

This study was funded by the following NIHR Health Protection Research Units (HPRU) in partnership with UK Health Security Agency (UKHSA): the Behavioural Science and Evaluation HPRU at University of Bristol (HPRU BSE), (Grant number: NIHR200877) and the Environmental Change and Health HPRU at London School of Health and Tropical Medicine and University College London (HPRU ECH), (Grant number: NIHR200909). The funding source had no role in review design, the collection, analysis and interpretation of data, the writing of the article or the decision to submit it for publication.

## Conflict of interest

The authors declare that the research was conducted in the absence of any commercial or financial relationships that could be construed as a potential conflict of interest.

## Publisher's note

All claims expressed in this article are solely those of the authors and do not necessarily represent those of their affiliated organizations, or those of the publisher, the editors and the reviewers. Any product that may be evaluated in this article, or claim that may be made by its manufacturer, is not guaranteed or endorsed by the publisher.

## Author disclaimer

The views expressed are those of the author(s) and not necessarily those of the NIHR, the Department of Health and Social Care, or UKHSA.
