# Supplementary material for: Factors associated with older adults' perception of health risks of hot and cold weather event exposure: A scoping review
Source: Front Public Health. 2022 Nov 10;10:939859. doi: 10.3389/fpubh.2022.939859 (PMC9686383; doi:10.3389/fpubh.2022.939859)
Supplement: Supplementary file 1 [file Table_1.DOCX]

**Search terms:**

***Context***-

- Risk perception
- Risk awareness
- Risk communication
- Risk behaviour
- Risk knowledge
- Risk practice
- Threat perception
- Threat awareness
- Threat communication
- Threat behaviour
- Threat knowledge
- Threat practice
- Alert
- Early Warning

***Threat/Risk****-*

- Heat wave/Heatwave
- Heat stress
- Heat strain
- Heat
- Heat spell
- Heat awareness
- Heat weather
- Heat episode
- Heat exposure
- Extreme heat
- Hot wave
- Hot spell
- Hot temperature
- Hot weather
- Hot episode
- Cold spell
- Cold
- Cold awareness
- Cold wave
- Cold Temperature
- Cold weather
- Cold episode
- Cold exposure
- Extreme cold
- Elevated temperature
- Reduced temperature
- Extreme temperature
