# Supplementary material for: Factors associated with older adults' perception of health risks of hot and cold weather event exposure: A scoping review
Source: Front Public Health. 2022 Nov 10;10:939859. doi: 10.3389/fpubh.2022.939859 (PMC9686383; doi:10.3389/fpubh.2022.939859)
Supplement: Supplementary file 2 [file Table_2.docx]

**APPENDIX 2**

**Database:**
Ovid MEDLINE(R) and Epub Ahead of Print, In-Process & Other Non-Indexed Citations and Daily

| **#** | **Query** | **Results from 2 Feb 2021** |
| --- | --- | --- |
| 1 | adaptation, psychological/ or attitude/ or behavior/ or accident proneness/ or exp health risk behaviors/ or exp information seeking behavior/ or exp risk reduction behavior/ | 186,255 |
| 2 | Health Knowledge, Attitudes, Practice/ | 114,921 |
| 3 | Health Communication/ | 2,569 |
| 4 | risk/ or protective factors/ | 128,958 |
| 5 | ((risk or threat) adj3 (perception* or perceive or communication* or aware* or attitude* or behavio$r* or knowledge or practice*)).tw. | 59,286 |
| 6 | alert.tw. | 26,427 |
| 7 | "early warning*".tw. | 7,104 |
| 8 | 1 or 2 or 3 or 4 or 5 or 6 or 7 | 501,865 |
| 9 | exp Hot Temperature/ | 118,221 |
| 10 | exp Cold Temperature/ | 74,928 |
| 11 | weather/ or exp temperature/ | 431,470 |
| 12 | ((heat or cold or hot) adj3 (wave* or stress* or strain or awareness or extreme* or spell or spells* or temperature or weather or snap or snaps or episode* or exposure*)).tw. | 46,763 |
| 13 | ((elevated or reduced or extreme*) adj3 temperature*).tw. | 25,640 |
| 14 | Heatwave*.tw. | 665 |
| 15 | Coldwave*.tw. | 2 |
| 16 | 9 or 10 or 11 or 12 or 13 or 14 or 15 | 470,564 |
| 17 | 8 and 16 | 2,088 |
| 18 | limit 17 to english language | 1,933 |

**Database:** Embase

| **#** | **Query** | **Results from 2 Feb 2021** |
| --- | --- | --- |
| 1 | exp high risk behavior/ | 28,698 |
| 2 | risk/ or exp population risk/ | 510,457 |
| 3 | exp life threat/ | 3,004 |
| 4 | ((risk or threat) adj3 (perception* or perceive or communication* or aware* or attitude* or behavio$r* or knowledge or practice*)).tw. | 74,074 |
| 5 | alert.tw. | 39,450 |
| 6 | "early warning*".tw. | 9,251 |
| 7 | 1 or 2 or 3 or 4 or 5 or 6 | 641,979 |
| 8 | heat/ or exp temperature related phenomena/ | 202,106 |
| 9 | exp cold/ | 23,113 |
| 10 | exp heat wave/ | 553 |
| 11 | extreme weather/ or exp extreme cold weather/ or exp extreme hot weather/ | 295 |
| 12 | ((heat or cold or hot) adj3 (wave* or stress* or strain or awareness or extreme* or spell or spells* or temperature or weather or snap or snaps or episode* or exposure*)).tw. | 50,766 |
| 13 | Heatwave*.tw. | 686 |
| 14 | Coldwave*.tw. | 1 |
| 15 | ((elevated or reduced or extreme*) adj3 temperature*).tw. | 25,946 |
| 16 | 8 or 9 or 10 or 11 or 12 or 13 or 14 or 15 | 249,251 |
| 17 | 7 and 16 | 2,935 |
| 18 | limit 17 to english language | 2,863 |

**Database:** APA PsycInfo

| **#** | **Query** | **Results from 2 Feb 2021** |
| --- | --- | --- |
| 1 | exp health risk behavior/ | 3,117 |
| 2 | exp risk perception/ or perception/ or hazards/ or prevention/ or prospect theory/ or risk taking/ or safety/ | 88,226 |
| 3 | threat/ or threat assessment/ | 11,089 |
| 4 | ((risk or threat) adj3 (perception* or perceive or communication* or aware* or attitude* or behavio$r* or knowledge or practice*)).tw. | 44,442 |
| 5 | alert.tw. | 6,179 |
| 6 | "early warning*".tw. | 919 |
| 7 | 1 or 2 or 3 or 4 or 5 or 6 | 137,723 |
| 8 | exp temperature effects/ | 4,979 |
| 9 | exp Heat Effects/ | 1,318 |
| 10 | exp atmospheric conditions/ | 4,117 |
| 11 | ((heat or cold or hot) adj3 (wave* or stress* or strain or awareness or extreme* or spell or spells* or temperature or weather or snap or snaps or episode* or exposure*)).tw. | 2,904 |
| 12 | ((elevated or reduced or extreme*) adj3 temperature*).tw. | 641 |
| 13 | Heatwave*.tw. | 29 |
| 14 | Coldwave*.tw. | 0 |
| 15 | 8 or 9 or 10 or 11 or 12 or 13 or 14 | 10,990 |
| 16 | 7 and 15 | 486 |
| 17 | limit 16 to english language | 480 |

**Database:** Web of Science

| **#** | **Query** | **Results from 2 Feb 2021** |
| --- | --- | --- |
| 1 | exp health risk behavior/ | 3,117 |
| 2 | exp risk perception/ or perception/ or hazards/ or prevention/ or prospect theory/ or risk taking/ or safety/ | 88,226 |
| 3 | threat/ or threat assessment/ | 11,089 |
| 4 | ((risk or threat) adj3 (perception* or perceive or communication* or aware* or attitude* or behavio$r* or knowledge or practice*)).tw. | 44,442 |
| 5 | alert.tw. | 6,179 |
| 6 | "early warning*".tw. | 919 |
| 7 | 1 or 2 or 3 or 4 or 5 or 6 | 137,723 |
| 8 | exp temperature effects/ | 4,979 |
| 9 | exp Heat Effects/ | 1,318 |
| 10 | exp atmospheric conditions/ | 4,117 |
| 11 | ((heat or cold or hot) adj3 (wave* or stress* or strain or awareness or extreme* or spell or spells* or temperature or weather or snap or snaps or episode* or exposure*)).tw. | 2,904 |
| 12 | ((elevated or reduced or extreme*) adj3 temperature*).tw. | 641 |
| 13 | Heatwave*.tw. | 29 |
| 14 | Coldwave*.tw. | 0 |
| 15 | 8 or 9 or 10 or 11 or 12 or 13 or 14 | 10,990 |
| 16 | 7 and 15 | 486 |
| 17 | limit 16 to english language | 480 |
